# Effect of Silane-Modified Nano-Al_2_O_3_-Reinforced Vinyl Ester Resin on the Flexural Properties of Basalt Fiber Composites

**DOI:** 10.3390/ma18081727

**Published:** 2025-04-10

**Authors:** Yuehai Wei, Yongda Miao, Leilei Ma, Wei Tian, Chenyan Zhu

**Affiliations:** 1 Key Laboratory of Advanced Textile Materials and Preparation Technology of the Ministry of Education, College of Textiles Science and Engineering, Zhejiang Sci-Tech University (Xiasha Campus), Hangzhou 310018, China; weiyuehai2000@163.com (Y.W.);; 2 State Key Laboratory of Bio-Based Fiber Materials, Zhejiang Sci-Tech University, Hangzhou 310018, China

**Keywords:** nano-alumina, silane coupling agent, vinyl ester resin, basalt fiber-reinforced polymer composites, flexural properties, elevated temperature aging

## Abstract

This study incorporated silane coupling agent KH550-modified nano-alumina (KH550-Al_2_O_3_) into vinyl ester resin (VER) for modification. The effect of KH550-Al_2_O_3_ on the flexural properties of VER and basalt fiber-reinforced vinyl ester resin (BF/VER) composites was investigated. In addition, dynamic mechanical analysis (DMA) and long-term elevated temperature aging of the composites were performed. The surface functionalization of KH550-Al_2_O_3_ was confirmed by Fourier transform infrared spectroscopy (FTIR), thermogravimetric analysis (TGA), and energy-dispersive X-ray spectroscopy (EDS). It was revealed by scanning electron microscopy (SEM) that the aggregation of KH550-Al_2_O_3_ had been reduced within the VER matrix, the resin was effectively enhanced, and the fiber–matrix interfacial bonding was improved. Based on the experimental results, the optimal filler loading of KH550-Al_2_O_3_ was 1.5 wt%. Compared with the control group, the resin matrix exhibited 18.1% and 22.7% improvements in flexural strength and modulus, respectively, while the composite showed increases of 9.3% and 7.6% in these properties. At 30 °C, the storage modulus of the composites increased by 11.5%, with the glass transition temperature rising from 111.0 °C to 112.5 °C. After 60 days of thermal aging at 120 °C, the retained flexural strength and modulus were 64.3% and 87.4%, respectively.

## 1. Introduction

Owing to their superior mechanical traits, durability, and environmental friendliness, basalt fiber-reinforced polymer matrix composites (BFRPs) find extensive applications in industries, including the automotive, sports, and construction sectors [1,2,3]. Basalt fiber (BF) has a higher elastic modulus and tensile strength than glass fiber and better environmental friendliness and cost-effectiveness than carbon fiber [4]. Therefore, compared with glass fiber or carbon fiber-reinforced resin-based composites, BFRP has comparable strength and modulus, and offers advantages such as abundant raw material sources, significant ecological benefits, and superior economic efficiency [5]. Three critical aspects determine the mechanical properties of BFRPs: the BF properties, the resin matrix properties, and the interfacial properties [6]. In BFRPs, the fiber–matrix interfacial bonding tends to be suboptimal due to BFs’ inherently low-surface-energy characteristics, insufficient interfacial contact area, and limited chemical reactivity [2].

Currently, two main methods are used to improve the interfacial bonding of BFRPs. One is the surface treatment of BF through methods such as coating [7], chemical grafting [8], plasma treatment [9], and in situ growth [10]. Another method is to modify the resin matrix by adding fillers [11,12,13]. Enhancing basalt fiber–resin interfacial bonding through surface treatment techniques is an effective approach for optimizing stress transfer efficiency. Nevertheless, the primary challenge lies in translating these strategies into viable processes for industrial-scale manufacturing [14]. The inherent flexural properties of the resin matrix significantly influence the integrated flexural behavior of the composites, while also dominating the out-of-plane mechanical behavior of laminated composites [15,16]. Furthermore, ambient temperature noticeably affects the resin matrix and its interfacial bonding with fibers [17]. When subjected to prolonged exposure in oxygen-containing elevated temperature environments, the resin matrix degrades due to thermal oxidation, leading to progressive embrittlement and a consequent deterioration in its mechanical properties [18,19]. Under elevated temperature environments, the disparities in the thermomechanical properties of the resin matrix and the reinforced fiber induce thermal stress within laminated composites, further leading to amplified microscale damage accumulation and, consequently, a reduction in interfacial bond strength [20,21,22]. One commonly used thermosetting resin in BFRPs is vinyl ester resin (VER) [1]. However, the inherent brittleness of VER renders basalt fiber-reinforced vinyl ester resin (BF/VER) composites prone to matrix crack initiation, which further leads to accelerated crack propagation under mechanical loading [23]. Therefore, enhancing the flexural properties of VER to improve the flexural properties of BF/VER composites demonstrates considerable application potential.

The uniform distribution of nanofillers into the thermosetting resin has been demonstrated to improve composite mechanical properties; this is achieved by enhancing the resin matrix and optimizing fiber–matrix interfacial compatibility, as evidenced by prior studies [23,24,25,26,27]. Among various inorganic nanofillers, nano-alumina (nano-Al_2_O_3_) has gained widespread application due to its superior thermal properties and cost-effective production characteristics [28]. The investigations of scholars have systematically characterized the reinforcement effects of nano-Al_2_O_3_ across various fiber-reinforced epoxy resin (EP) systems. Vinay et al. [28] identified optimal enhancement in flexural strength, impact strength, wear rate, and interlaminar shear for EP matrix composites reinforced with basalt fibers at specific nano-Al_2_O_3_ loadings. Kaybal et al. [29] demonstrated improvement in energy absorption capacity for EP matrix composites reinforced with carbon fibers under low-velocity impact by adding nano-Al_2_O_3_. Mahato et al. [30] revealed the temperature-dependent flexural behavior of EP matrix composites reinforced with glass fibers through gradient loadings of nano-Al_2_O_3_.

Despite exhibiting exceptional efficacy in augmenting the mechanical properties of composites, nano-Al_2_O_3_ particles are inherently prone to aggregation within the resin matrix due to their elevated surface energy, which is a direct consequence of their inherently high specific surface area [31,32]. The effectiveness of nanoparticle-reinforced resin matrices is predominantly governed by their dispersion homogeneity within the matrix [33]. Consequently, surface modification represents a viable strategy to mitigate nanoparticle aggregation and optimize their dispersion uniformity within the resin matrix [14,34,35]. The silane coupling agent KH550 is widely utilized as a surface modifier for fillers in resin matrix composites, effectively enabling better integration of nanoparticles into the resin systems and promoting dispersion [36,37,38]. Li et al. [39] confirmed that the KH550-modified nano-zirconium dioxide (nano-ZrO_2_) exhibited improved dispersion in EP, significantly enhancing the fracture toughness and tensile strength of EP. Sun et al. [40] further developed a dual-modification protocol combining KH550 with polyvinylpyrrolidone (PVP) nano-ferric oxide (nano-Fe_2_O_3_), achieving superior mechanical enhancement compared with singular PVP treatment. While typical VER exhibits relatively inferior mechanical properties compared with high-performance EP, it presents a competitive alternative due to its cost-effective manufacturability, tunable curing kinetics, and simplified curing protocols [41,42,43]. Nevertheless, when exploring the effect of nano-alumina on the mechanical properties of resin or fiber-reinforced resin-based composite materials, previous studies have mostly focused on a single-material scale (resin matrix or composites) [32,44,45], and the complete experimental chain from resin reinforcement to composite material performance improvement still needs to be systematically revealed. Meanwhile, existing research efforts have predominantly focused on EP systems, with limited investigations dedicated to VER modification through KH550-modified nano-Al_2_O_3_ (KH550-Al_2_O_3_), particularly regarding the specific effects of such modified nanoparticles on the flexural properties of BF/VER composites.

This study systematically investigated the influence of KH550-Al_2_O_3_ on the flexural properties of VER and BF/VER composites, elucidating the interfacial reinforcement mechanisms through in-depth analysis of VER properties. Surface functionalization of nano-Al_2_O_3_ with KH550 was verified through Fourier transform infrared spectroscopy (FTIR), thermogravimetric analysis (TGA), and energy-dispersive X-ray spectroscopy (EDS). The correlation between KH550-Al_2_O_3_ and the flexural properties of the resins and their composites was established via three-point bending tests, two-way analysis of variance (ANOVA), and fracture surface characterization using scanning electron microscopy (SEM). The viscoelastic behavior of the composites was evaluated through dynamic mechanical analysis (DMA). Finally, the flexural properties of the composites after elevated-temperature aging were also assessed. Consequently, this study used KH550-Al_2_O_3_ as the filler and simultaneously optimized the flexural properties of the BF/VER composites under normal conditions and after long-term elevated temperatures, as well as dynamic mechanical properties, for potential utilization in engineering systems.

## 2. Materials and Methods

### 2.1. Materials

Nano-Al_2_O_3_ (α-phase, average particle size of 30 nm), silane coupling agent KH550 (3-aminopropyltriethoxysilane, 99% purity), and glacial acetic acid (reagent grade) were purchased from Shanghai Macklin Biochemistry Technology Co., Ltd. (Shanghai, China). Bisphenol A-type vinyl ester resin (solid content of 55 ± 1%, SWANCOR 901) and the accelerator (cobalt content of 1.6%, SWANCOR 1305) were purchased from Swancor Advanced Materials Co., Ltd. (Shanghai, China). The curing agent (Trigonox V388, active oxygen content of 9.8–10%) was purchased from Nouryon Chemicals (Ningbo) Co., Ltd. (Ningbo, China). Absolute ethanol, deionized water, and polyimide film (for the mold release of the composites) were purchased from Hangzhou Gaojing Fine Chemical Industry Co., Ltd. (Hangzhou, China). Basalt fiber plain fabrics (BF, unit weight of 300 g/m^2^, 50 ends/10 cm of warp density and weft density) were purchased from Haining Anjie Composite Materials Co., Ltd. (Jiaxing, China).

### 2.2. Surface Modification of Nano-Al_2_O_3_

**Figure 1:**
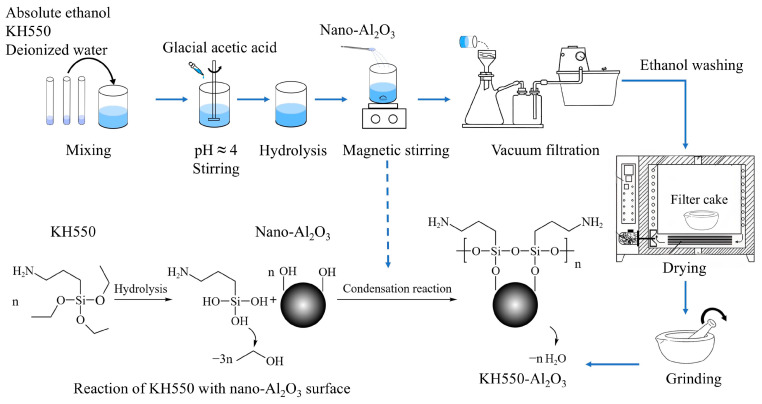
Schematic diagram of the KH550 surface-modified nano-Al_2_O_3_ process.

As shown in Figure 1, the essence of KH550 surface-modified nano-Al_2_O_3_ is as follows: first, KH550 hydrolyzes into silanol intermediates, which then react with hydroxyl groups on nano-Al_2_O_3_ surfaces through a condensation reaction, thereby self-assembling into polysiloxane networks [46]. The experimental findings of Brochier et al. [47] revealed that silanol groups formed through KH550 hydrolysis exhibit notable stability in acidic environments, demonstrate minimal self-condensation propensity, and achieve optimal hydrolysis efficiency when the silane concentration reaches 10% (*w*/*w*) relative to the solvent. Therefore, the process of KH550 surface-modified nano-Al_2_O_3_ in this study was conducted as follows.

We measured 10 mL of KH550, 30 mL of deionized water, and 60 mL of absolute ethanol. Following this, absolute ethanol, KH550, and deionized water were added successively and slowly to the beaker while stirring after each addition (the concentration of KH550 was about 10.9%). The mixed solution was acidified to a pH of about 4 through the dropwise addition of glacial acetic acid, followed by static incubation at ambient temperature for 1 h to complete the reaction. Subsequently, 10 g of dried nano-Al_2_O_3_ was added to the above mixed solution. The mouth of the beaker was sealed with a transparent film, and the reaction was magnetically stirred at 45 °C for 500 r/min for 6 h. The post-reaction mixture underwent vacuum filtration to isolate the solid phase. Following the completion of the reaction, the resulting mixture was subjected to vacuum filtration. The retained filter cake underwent multiple washes with absolute ethanol. Subsequently, the product was dried at a temperature of 80 °C for a duration of 12 h. Finally, the dried material was ground to produce KH550-Al_2_O_3_.

### 2.3. Preparation of Modified VERs

**Figure 2:**
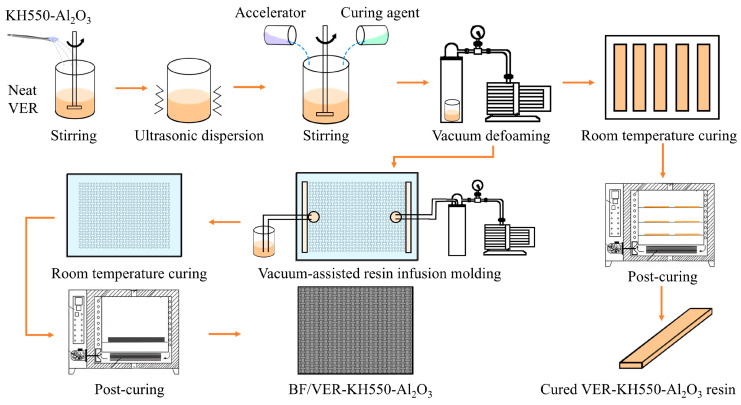
Schematic diagram of the process of KH550-Al_2_O_3_ modified VER and its composites.

We added a certain amount of KH550-Al_2_O_3_ to the vinyl ester resins (VERs). Initially, the resin blend was agitated mechanically in a 40 °C thermostatically controlled water bath for 30 min, followed by ultrasonic dispersion for 1 h. After the resin blend was cooled to room temperature, according to the mass ratio of VER–accelerator–curing agent = 100:1:2, we added the accelerator to the mixture first, then added the curing agent, and stirred well after adding each component. Subsequently, the air bubbles in the resin blend were eliminated under a vacuum and then transferred to a pre-prepared mold of a specific size. The resin blend was cured at room temperature for 24 h, followed by post-curing in a 100 °C oven for 2 h. Final cooling to room temperature and demolding yielded the modified VERs (Figure 2). In parallel, the neat VER and the VER with unmodified nano-Al_2_O_3_ as filler were prepared under identical conditions. The cured specimens used for testing were named Neat VER (blank control group), VER-Al_2_O_3_ x wt% (the VER with unmodified nano-Al_2_O_3_ as filler), and VER-KH550-Al_2_O_3_ x wt% (the VER with KH550-Al_2_O_3_ as filler), where “x wt%” denotes the weight percentage of filler relative to resin.

### 2.4. Preparation of BF/VER Composites

The BF/VER composites were fabricated using the vacuum-assisted resin infusion molding (VARIM) process. The materials preparation process involved the following steps: First, a layered structure was assembled in a bottom-to-top sequence containing a flat plate, one polyimide film layer, eight ethanol-cleaned and dried basalt fiber plain woven fabric layers (oriented at 0°), one release film layer, and one resin distribution medium layer. This assembly was then positioned on a vacuum bag membrane bordered by sealant tape, ensuring the sealed area exceeded the plate dimensions. The guide tube and injection port were secured on opposite sides of the deflector mesh. A vacuum bag film was then applied to encapsulate the assembly, with a pinhole created in the film above the injection port center. The hose was carefully inserted into the injection port, with the opposite end of the hose connected to a vacuum pump. Following activation, the system’s airtightness was verified through fabric compaction under negative pressure. Following the procedure outlined in Section 2.3, the degassed resin blend was introduced through the opposite hose. After complete resin impregnation and uniform distribution through the basalt fiber fabrics, vacuum maintenance continued for 10 min. The prepreg laminate was subsequently cured at room temperature for 24 h, followed by post-curing in a 100 °C oven for 2 h. Final cooling to room temperature yielded the composites. In parallel, the composites with the neat VER as the matrix were prepared under identical conditions. The specimens used for testing were named BF/VER-control (blank control group) and BF/VER-KH550-Al_2_O_3_ y wt% (basalt fiber-reinforced vinyl ester resin composites with KH550-Al_2_O_3_ as filler), where “y wt%” denotes the weight percentage of filler relative to resin.

### 2.5. Elevated Temperature Aging of BF/VER Composites

Owing to the laminated composites being exposed to temperatures exceeding their glass transition temperature (T_g_), their mechanical properties often significantly decreased [48]. To reduce the potential impact caused by changes in T_g_, the aging temperature of composite materials was set to exceed their T_g_. The BF/VER composites were aged within the high- and low-temperature test chamber (BPH-060C, Shanghai Yiheng Technology, Shanghai, China). The control BF/VER composites and optimized BF/VER-KH550-Al_2_O_3_ composites were machined to dimensions that complied with mechanical testing standards. Specimens underwent surface cleaning with anhydrous ethanol, followed by 24 h of conditioning at 23 °C in an electric blast oven. Accelerated aging involved four experimental groups per material exposed to 120 °C thermal aging for progressive durations: 15, 30, 45, and 60 days, ultimately yielding eight test groups (2 materials × 4 aging periods).

### 2.6. Characterization

#### 2.6.1. Fourier Transform Infrared Spectrum Tests

Fourier transform infrared spectroscopy (Nicolet iS50, Thermo Fisher Scientific, Waltham, MA, USA) was used to analyze the specimens via infrared spectroscopy with a scanning resolution of 4 cm^−1^ and 32 scans, providing information on the characteristic functional groups of the specimen in the mid-infrared region. The samples, including KH550, unmodified nano-Al_2_O_3_, and KH550-Al_2_O_3_, were examined using potassium bromide (KBr) tableting, while the composites were analyzed with the attenuated total reflection (ATR) method.

#### 2.6.2. Thermogravimetric Tests

TGA tests were performed on unmodified nano-Al_2_O_3_ and KH550-Al_2_O_3_ using the synchronous thermal analyzer (STA 2500 Regulus, NETZSCH, Selb, Germany) to assess and verify the surface modification of nano-Al_2_O_3_ with KH550. The experiments were conducted in a nitrogen environment with a temperature range of 20 °C to 800 °C, employing a heating rate of 10 °C per minute. In addition, TGA tests on the cured resins were carried out using the thermogravimetric analyzer (TGA 550, TA Instruments, New Castle, DE, USA) to evaluate the impact of KH550-Al_2_O_3_ on the thermal stability of VER. The experiments were conducted in an air environment with a temperature range of 25 °C to 800 °C, employing a heating rate of 10 °C per minute.

#### 2.6.3. Three-Point Bending Tests

**Figure 3:**
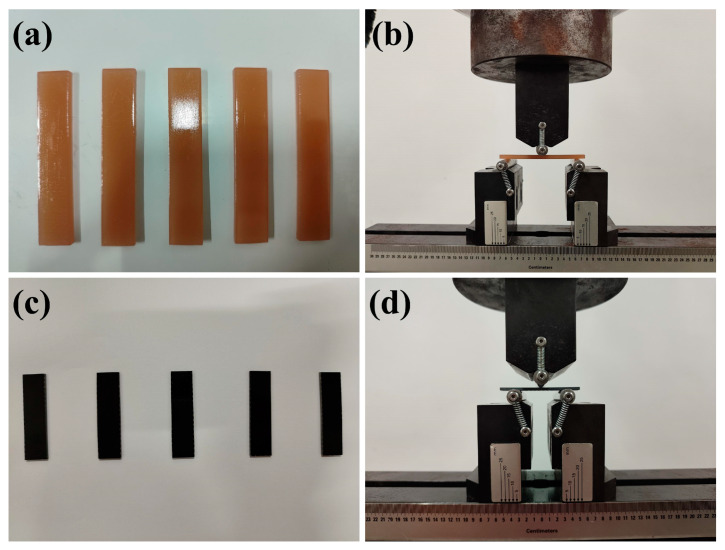
Three-point bending test diagrams: (**a**) resin specimens, (**b**) resin specimens under load, (**c**) composite specimens, and (**d**) composite specimens under load.

Three-point bending tests were performed using the universal mechanical testing machine (Landmark 370.10, MTS Systems, Eden Prairie, MN, USA) to evaluate the impact of filler on the flexural properties of specimens, as illustrated in Figure 3. Each group consisted of five specimens, and the data were averaged for analysis. The resin’s flexural properties were evaluated according to Chinese National Standard GB/T 2567-2021 [49], using specimens with dimensions of 80 mm × 15 mm × 4 mm with a testing span of 64 mm and a loading rate of 2 mm/min. Composite flexural properties were measured in compliance with Chinese National Standard GB/T 1449-2005 [50], using specimens with dimensions of 60 mm × 15 mm × 2 mm with a testing span of 32 mm and a loading rate of 2 mm/min.

The flexural strength of specimens was calculated using the following formula:

(1) $$\sigma_{f}=\frac{3P\cdot L}{2b\cdot h^{2}}$$

where $\sigma_{f}$ is the flexural strength (MPa), P is the breaking load (N), L is the span (mm), b is the specimen width (mm), and h is the specimen thickness (mm).

The flexural strain of specimens was calculated using the following formula:

(2) $$\epsilon_{f}=\frac{6S\cdot h}{L^{2}}\times 100$$

where $\epsilon_{f}$ is the flexural strain (%) and S is the deflection (mm) at the midpoint of the span corresponding. L and h are the same as in Formula (1).

The flexural modulus of specimens was calculated using the following formula:

(3) $$E_{f}=\frac{L^{3}\cdot ∆P}{4b\cdot h^{3}\cdot ∆S}$$

where $E_{f}$ is the flexural modulus (MPa), ∆P is the load increment (N) at the initial straight-line segment on the load–deflection curve, and ∆S is the deflection increment (mm) at the midpoint of the span corresponding to ∆P. L, b, and h are the same as in Formula (1).

#### 2.6.4. Scanning Electron Microscope Imaging

The scanning electron microscope (JSM-5610LV, JEOL, Tokyo, Japan) was used to capture images of the fracture section of the VER, the BF/VER composites, and the aged BF/VER composites. This analysis aimed to elucidate the failure modes of different materials and assess the interfacial bonding. In addition, an energy-dispersive spectrometer (INCA Energy, Oxford Instruments, Oxford, UK) was used to analyze the surface element distribution of KH550-Al_2_O_3_. The gold spraying treatments of the specimens were all performed using an ion sputtering apparatus with a gold spraying time of 2 min.

#### 2.6.5. Dynamic Mechanical Analysis Tests

The dynamic thermomechanical analyzer (DMA 1, METTLER TOLEDO, Columbus, OH, USA) was used to conduct the DMA tests on the BF/VER composites. This analysis aimed to evaluate the impact of KH550-Al_2_O_3_ on the viscoelastic properties of the composites and ascertain the glass transition temperature (T_g_). DMA was conducted in the three-point bending configuration using specimens measuring 30 mm × 10 mm × 2 mm, with testing parameters set to 1 Hz frequency, 10 μm amplitude, and a temperature range of 30 °C to 215 °C, employing a heating rate of 5 °C per minute, which was maintained under ambient air conditions.

## 3. Results

### 3.1. Characterization of KH550-Al_2_O_3_

**Figure 4:**
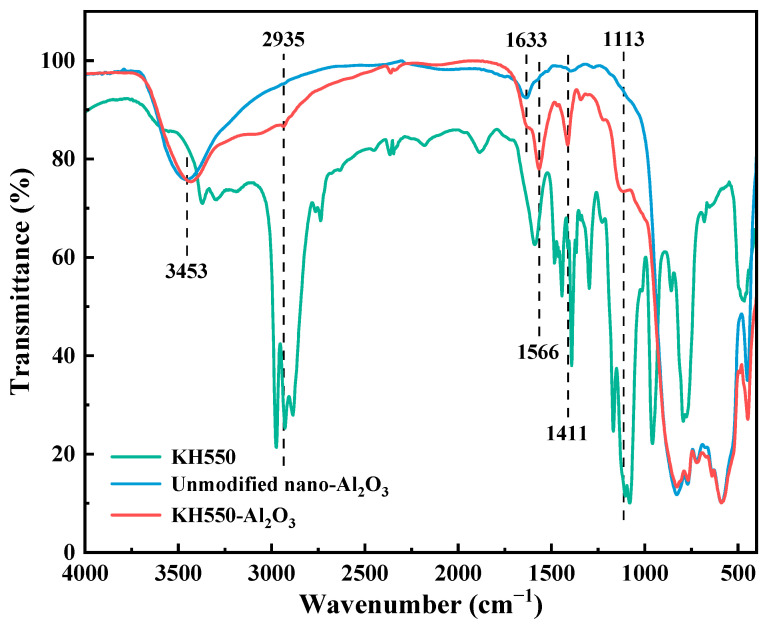
The infrared spectra of KH550, unmodified nano-Al_2_O_3_, and KH550-Al_2_O_3_.

FTIR was employed to analyze the chemical structures of KH550, unmodified nano-Al_2_O_3_, and KH550-Al_2_O_3_. The corresponding FTIR results are presented in Figure 4. For unmodified nano-Al_2_O_3_, O-H stretching vibration at 3453 cm^−1^ and H-O-H bending vibration at 1633 cm^−1^ are observed in its infrared spectrum, respectively, which demonstrates the presence of hydroxyl groups and adsorbed water on the surface of nano-Al_2_O_3_. In marked contrast, the spectrum of KH550-Al_2_O_3_ reveals several distinct peaks: C-H stretching vibration from methylene at 2935 cm^−1^, N-H bending vibration from primary amines at 1566 cm^−1^, C-H bending vibration from methylene adjacent to silicon at 1411 cm^−1^ [51], and a Si-O stretching vibration peak at 1113 cm^−1^ [52]. These peaks are absent in the unmodified nano-Al_2_O_3_ spectrum at the corresponding wavenumbers, confirming the presence of KH550 on the surface of KH550-Al_2_O_3_. Notably, the absorption peak intensity of KH550-Al_2_O_3_ around 3431 cm^−1^ does not show significant changes compared with the unmodified nano-Al_2_O_3_. This may be due to the reduction in hydroxyl groups on the surface of the nano-Al_2_O_3_ reaction, while the N-H stretching vibration of the amino groups and the O-H stretching vibration in the unreacted silanol groups overlap here. The combined effect of these two factors ultimately leads to the observed spectral features.

However, KH550 attached to the surface of nano-Al_2_O_3_ by hydrogen bonding is much less thermally stable than KH550 attached by covalent bonding and breaks down at comparatively lower temperatures [53]. Therefore, to determine the presence of KH550 connected by covalent bonds on the surface of KH550-Al_2_O_3_, TGA tests were performed on unmodified nano-Al_2_O_3_ and KH550-Al_2_O_3_.

**Figure 5:**
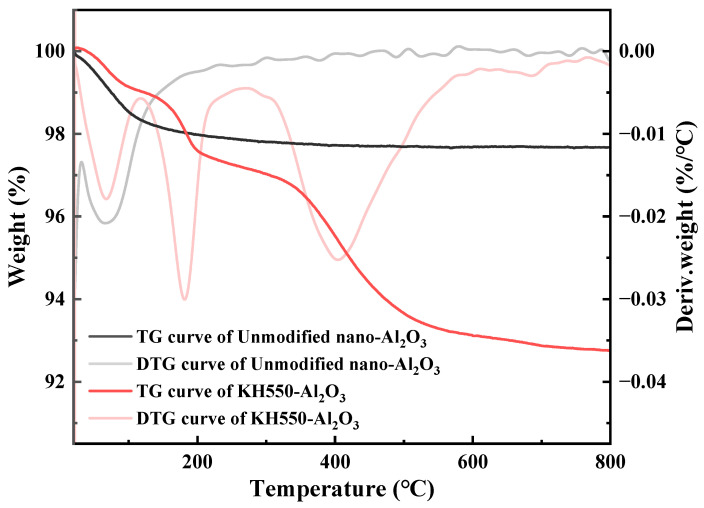
TGA curves of unmodified nano-Al_2_O_3_ and KH550-Al_2_O_3_ in nitrogen.

Figure 5 shows the thermogravimetric (TG) and derivative thermogravimetric (DTG) profiles of unmodified nano-Al_2_O_3_ and KH550-Al_2_O_3_ under nitrogen. For unmodified nano-Al_2_O_3_, only one mass loss phase occurred throughout the test, which was attributable to the desorption of adsorbed water and the breakdown of hydroxyl groups. In contrast, KH550-Al_2_O_3_ exhibited three mass loss phases across the tested temperature range. The first mass loss phase of KH550-Al_2_O_3_ occurred before 120 °C due to the desorption of adsorbed water. Within this phase, KH550-Al_2_O_3_ demonstrated a lower mass loss rate compared with unmodified nano-Al_2_O_3_, which suggests that KH550 occupied a certain number of active sites on the surface of nano-Al_2_O_3_, thereby suppressing moisture adsorption. The second mass loss phase, occurring from 120 °C to 270 °C, was primarily associated with the decomposition of both residual KH550 bound via hydrogen bonding to the nano-Al_2_O_3_ surfaces and unreacted hydroxyl groups that remained on nano-Al_2_O_3_ surfaces during the condensation process. The third mass loss phase, occurring after 270 °C, was primarily associated with the decomposition of KH550 bonded to the surface of nano-Al_2_O_3_ via covalent bonding. Therefore, the results from FTIR and TGA collectively confirm the grafting of KH550 as polysiloxanes onto nano-Al_2_O_3_ surfaces, while indicating the presence of excess siloxane residues.

**Figure 6:**
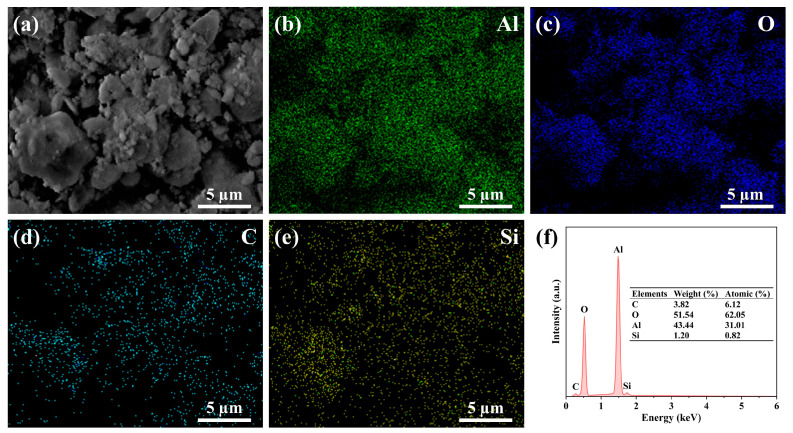
(**a**) SEM image of KH550-Al_2_O_3_, (**b**–**e**) elemental mapping of Al, O, C, and Si, and (**f**) EDS analysis of KH550-Al_2_O_3_.

EDS was employed to analyze the KH550-Al_2_O_3_ surface composition. The corresponding EDS results are presented in Figure 6. While EDS provides semi-quantitative data, it effectively characterizes elemental distribution. The spatial distributions of Al, O, C, and Si elements demonstrate remarkable consistency, further substantiating the presence of KH550 on the nano-Al_2_O_3_ surface. Notably, although FTIR shows the presence of amino groups, EDS fails to detect elemental nitrogen. This phenomenon likely arises from a barrier of excessive polysiloxane on nano-Al_2_O_3_ surfaces combined with strong absorption of low-concentration N-Kα radiation (0.392 keV) of Al_2_O_3_. At such minimal levels, this absorption causes signal attenuation below EDS detection thresholds. A similar observation is noted in the study of KH550 surface-modified filler, where a high mass ratio of KH550 to the modified filler is present. Despite this, the EDS analysis reveals non-existent or extremely low nitrogen content in the modified material [54,55,56].

### 3.2. Flexural Properties of VERs

**Figure 7:**
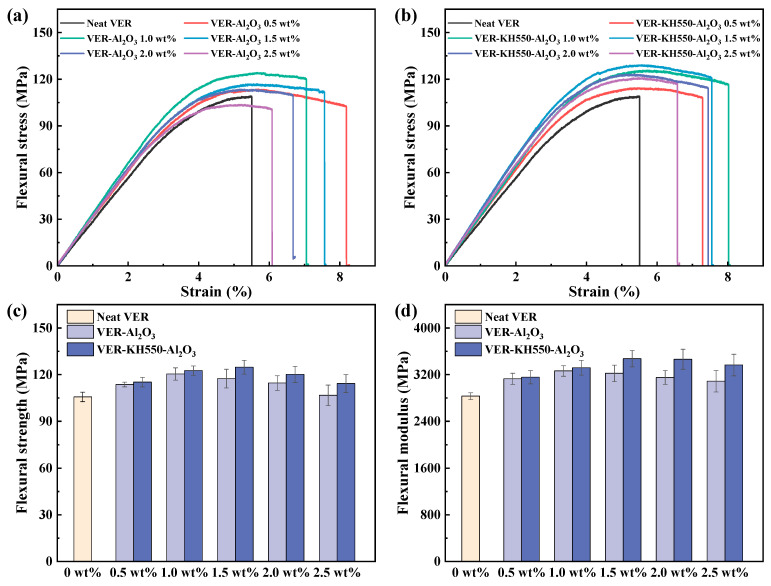
Flexural properties of VERs added with unmodified nano-Al_2_O_3_ and KH550-Al_2_O_3_ at different filler loads: (**a**) VER-Al_2_O_3_ resin’s flexural stress–strain curves, (**b**) VER-KH550-Al_2_O_3_ resin’s flexural stress–strain curves, (**c**) flexural strength, and (**d**) flexural modulus.

Figure 7 illustrates the influence of filler loading of unmodified nano-Al_2_O_3_ and KH550-Al_2_O_3_ on the flexural properties of the VERs. Figure 7a,b illustrate the typical three-point bending stress–strain curves for the VERs. All the resins containing either unmodified nano-Al_2_O_3_ or KH550-Al_2_O_3_ have significantly larger areas under the curve when benchmarked against the neat resin. This observation indicates that the incorporation of nano-Al_2_O_3_ effectively promotes the energy absorption of the resin before fracture. The VER-Al_2_O_3_ resin achieved maximum fracture strain at 0.5 wt% filler loading, while the VER-KH550-Al_2_O_3_ resin achieved maximum fracture strain at 1.0 wt% filler loading. Beyond these levels, both resin systems demonstrated progressively reduced fracture strain with increasing filler loading, while the VER-Al_2_O_3_ resin showed a relatively greater decrease in the strain value at fracture. One end of KH550 is grafted on the surface of nano-Al_2_O_3_ with Si-O-Al bonds, while the other end can be connected to the molecular chain of VER through amino groups [57]. The formation of this double-ended chemical bonding between nano-Al_2_O_3_ and VER increases the compatibility between them, retarding the negative effects caused by excess fillers on the resin. As a result, with increasing filler loading, the unmodified nano-Al_2_O_3_ induced relatively premature fracture of the resin under load owing to agglomeration.

Figure 7c,d illustrate the effects of filler loading on the flexural strength and flexural modulus of the VERs. The flexural properties of all the resins containing either unmodified nano-Al_2_O_3_ or KH550-Al_2_O_3_ have improved to different degrees. The flexural strength and flexural modulus of the neat VER measured 105.7 MPa and 2830.2 MPa, respectively. At 1.5 wt% filler loading, the VER-Al_2_O_3_ resin demonstrated enhanced flexural strength and flexural modulus to 117.4 MPa and 3222.2 MPa, reflecting enhancements of 11.1% and 13.9% when benchmarked against the neat resin. In contrast, the VER-KH550-Al_2_O_3_ resin exhibited the maximum flexural strength of 124.8 MPa and modulus of 3472.7 MPa, respectively, representing increases of 18.1% and 22.7% for the same filler load. Nonetheless, whether or not nano-Al_2_O_3_ was modified did not appear to produce a pronounced impact on the flexural properties of VERs due to the overlap of the error intervals in the data. Therefore, a two-factor ANOVA was conducted to evaluate the impact of nano-Al_2_O_3_ surface modification and its loading on the flexural properties of the VERs.

Table 1 and Table 2 show the results of two-way ANOVA for flexural strength and flexural modulus of the modified resin. The results show that for the flexural strength and flexural modulus of VER within the range of filler loading investigated, the main effects of whether or not the nano-Al_2_O_3_ was modified and its loading were statistically significant (Sig. < 0.05), indicating that the modification treatment and filler loading independently affect the flexural properties of the resins. Specifically, the surface modification treatment of KH550 enhanced the flexural properties of VER by improving the compatibility between nano-alumina and VER, while the increase in filler loading systematically affected the flexural properties of VER either through the enhancement mechanism of nanoparticles or the stress transfer effect. Notably, the interaction between the two factors did not reach the level of significance for the flexural strength (Sig. = 0.452) or flexural modulus (Sig. = 0.080) of VER, suggesting that the effect of the modified treatment was not significant under different filler loads and that the mechanism of the filler loading on flexural properties of VER did not change significantly depending on whether or not they were modified. Meanwhile, the flexural properties of VERs containing unmodified nano-Al_2_O_3_ or KH550-Al_2_O_3_ decreased gradually as the filler loading increased. Therefore, SEM was employed to examine resin fracture surfaces, elucidating the reinforcement mechanisms and actual dispersion state of nano-Al_2_O_3_ within VER.

**Figure 8:**
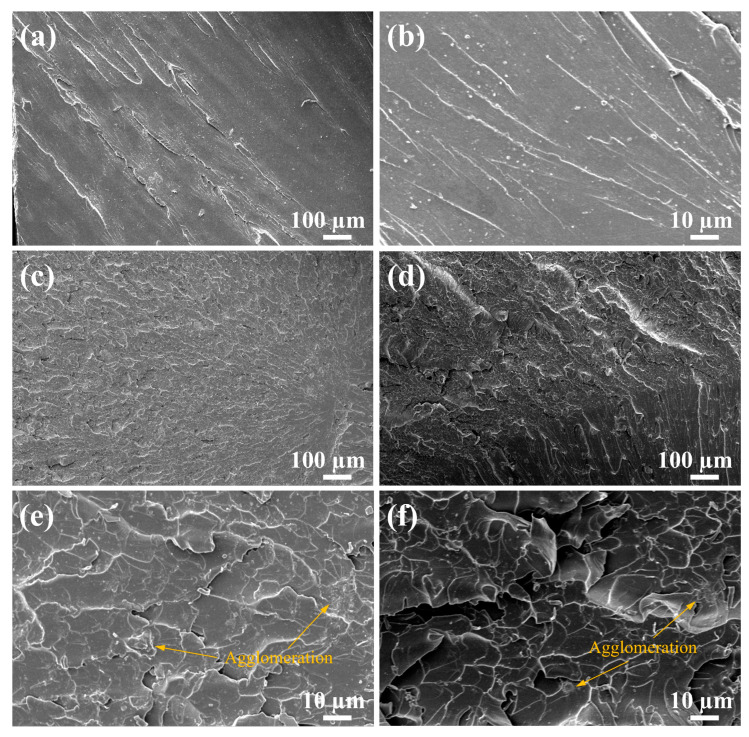
SEM images of the fracture section of the VERs after the tests: (**a**) neat VER at 100× magnification, (**b**) neat VER at 1000× magnification, (**c**) VER-Al_2_O_3_ 1.5 wt% resin at 100× magnification, (**d**) VER-KH550-Al_2_O_3_ 1.5 wt% resin 100× magnification, (**e**) VER-Al_2_O_3_ 1.5 wt% resin at 1000× magnification, and (**f**) VER-Al_2_O_3_ 1.5 wt% resin at 1000× magnification.

Resin fracture typically originates from defect sites, with crack sequential propagation generating three distinct characteristic zones on fracture surfaces: “mirror”, “mist”, and “hackle”. The hackle zone, which contains dense crack networks, reflects fracture energy dissipation mechanisms and the situation during crack evolution [58]. Figure 8 presents the fracture section of the VER specimens after the tests. The neat VER displayed a relatively flat morphology (Figure 8a) in the hackle zone with linear crack propagation and directional feather-like microcracks (Figure 8b), which was indicative of a brittle fracture of the neat resin due to the inability to dissipate the fracture energy effectively through self-deformation [59]. At 1.5 wt% unmodified nano-Al_2_O_3_ filler loading, the hackle zone in the VER became rough, accompanied by tortuous crack paths (Figure 8c). This indicates that the incorporation of unmodified nano-Al_2_O_3_ deflected and bifurcated the crack extension in the matrix, which facilitated the energy dissipation during fracture propagation. In contrast, at 1.5 wt% KH550-Al_2_O_3_ filler loading, the VER exhibited a coarser morphology featuring increased fracture surfaces and irregular crack patterns (as shown in Figure 8d), confirming that silane functionalization improved the adhesion of nano-Al_2_O_3_ to VER, resulting in intensified resin deformation and thus consuming more fracture energy.

SEM images at 1000× magnification identified that agglomeration was observed in both resin systems with fillers, with larger agglomeration noted by the orange arrows in the figure. The fracture surface of the VER-Al_2_O_3_ 1.5 wt% resin exhibited agglomeration aligned along crack propagation paths (Figure 8e), whereas the VER-KH550-Al_2_O_3_ 1.5 wt% resin displayed fewer and smaller instances of agglomeration (Figure 8f), which indicates that KH550-Al_2_O_3_ had greater compatibility in VER relative to unmodified nano-Al_2_O_3_. The mechanical properties of the resin-containing nanofillers exhibited significant sensitivity to filler agglomeration. Sukur et al. [60] indicated that sub-5 μm filler agglomeration demonstrates beneficial crack-arresting functionality to the resin matrix through mechanical reinforcement effects. This relatively small agglomeration effectively inhibits crack propagation by modifying stress distribution patterns within the resin matrix. Conversely, a relatively large amount of agglomeration induces interfacial discontinuity between nanofillers and resin matrix, creating localized stress concentration zones that compromise material integrity. Such morphological imperfections serve as preferential sites for fracture initiation, ultimately diminishing the structural integrity and energy dissipation capacity of the resin under mechanical loading.

In summary, crack deflection and crack pinning are observed as predominant reinforcement mechanisms of nano-Al_2_O_3_ in the VERs. When nano-Al_2_O_3_ particles are dispersed in the resin matrix, they effectively hinder crack propagation. Specifically, when a crack propagates near the nano-Al_2_O_3_ particles, its advancement is temporarily arrested. At this point, the crack can propagate in two main ways. One is by debonding along the interface between the nano-Al_2_O_3_ particles and the resin matrix, causing the original crack to branch into new, smaller cracks. The other involves a forced change in propagation direction, bypassing the nano-Al_2_O_3_ particles and forming a tortuous path. However, crack propagation along the interface debonding requires overcoming the bond strength between the nano-Al_2_O_3_ particles and the resin matrix, while the tortuous propagation path increases the overall length of crack propagation, both of which make crack propagation more difficult. Due to the large surface activity of nanoparticles, they can continuously consume the crack energy generated by fracture through numerous interfacial interactions.

For KH550-Al_2_O_3_, on the one hand, the compatibility between the nano-Al_2_O_3_ particles and the resin matrix is improved to a certain extent via the formation of double-end chemical bonds after surface modification, making crack propagation more difficult in the two aforementioned ways. On the other hand, excessive silane can lead to the encapsulation of nano-Al_2_O_3_ surfaces by an overly thick polysiloxane layer. The additional organic layer will form a weak interface between a portion of nano-Al_2_O_3_ and the resin matrix, increasing the possibility of slippage between them [61]. When the filler loading increases, the increase in slippage weakens the positive effect of the surface modification. Therefore, although the agglomeration of KH550-Al_2_O_3_ in VER has been reduced, the actual improvement in flexural properties of VER-KH550-Al_2_O_3_ resin is not significant compared with VER-Al_2_O_3_ resin.

### 3.3. Flexural Properties of BF/VER Composites

**Figure 9:**
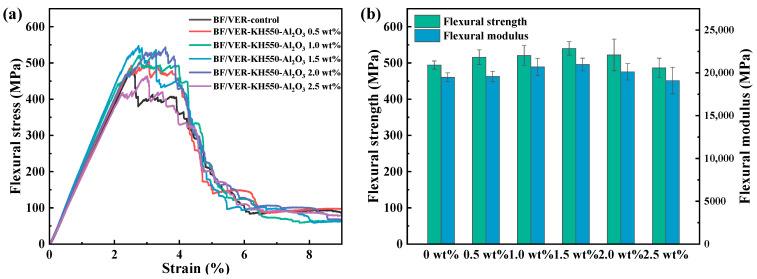
Flexural properties of the BF/VER composites with added KH550-Al_2_O_3_ in different filler loads: (**a**) flexural stress–strain curves and (**b**) flexural strength and flexural modulus.

Figure 9 illustrates the influence of KH550-Al_2_O_3_ filler loading on the flexural properties of the BF/VER composites. Figure 9a displays the typical three-point bending stress–strain curves for the BF/VER composites containing varying KH550-Al_2_O_3_ filler loads. All the composites displayed nonlinear elastoplastic behavior during bending. The control composites demonstrated an abrupt stress reduction following the initial linear phase, with rapid post-peak decay, indicating higher brittle fracture propensity. With the addition of KH550-Al_2_O_3_, the BF/VER composites showed higher stresses before failure and higher strains when the peak stresses were reached, which indicated that the presence of KH550-Al_2_O_3_ effectively improved the load-bearing capacity of the BF/VER composites. At 2.0 wt% filler loading, inside and outside the linear elastic region exhibited three distinct changes: decreased slope, extended stress fluctuations before peak stress, and increased strain at maximum stress. These observations indicate that, for this filler load, while the BF/VER composites demonstrated improved load-bearing capacity, the intensification of filler agglomeration concurrently compromised the interfacial adhesion between the reinforcing fibers and the resin matrix to some extent. The weakened interface reduced the bending load transfer efficiency between the resin matrix and reinforcement fibers, leading to a stiffness decline. Furthermore, when the filler load increased to 2.5 wt%, the load-bearing capacity of the BF/VER composites dropped significantly.

Figure 9b illustrates the influence of filler loading on the flexural strength and flexural modulus of the BF/VER composites. At 1.5 wt% filler load, the composites achieved peak flexural strength of 540.3 MPa and modulus of 20,978.2 MPa, respectively. Compared with the control BF/VER composites, which had a flexural strength of 494.4 MPa and a flexural modulus of 19,489.3 MPa, these values represent increases of 9.3% and 7.6%. However, as the filler load increased beyond this point, both the flexural strength and modulus began to decline, accompanied by notable data scatter. At 2.5 wt% filler load, these properties decreased to levels below those of the control composites. This trend indicates that enhanced filler agglomeration contributes to additional local stress concentration areas, disrupting the continuity between the fibers and the resin matrix and leading to an increase in defects within the composites. These defects are more prone to cause the growth and propagation of cracks during damage, ultimately resulting in material failure. In other words, the flexural properties of the BF/VER composites with KH550-Al_2_O_3_ are more sensitive to filler agglomeration.

**Figure 10:**
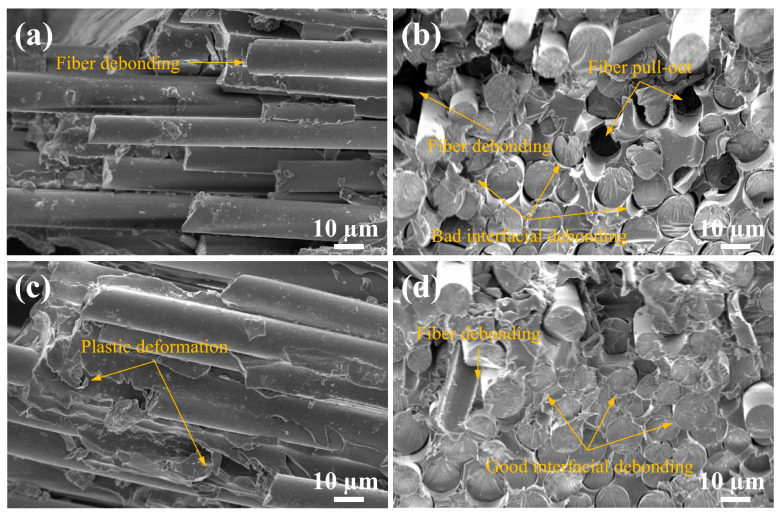
SEM images of the fracture section of the BF/VER composites after the tests: (**a**,**b**) the BF/VER-control composites at 1000× magnification; (**c**,**d**) the BF/VER-KH550-Al_2_O_3_ 1.5 wt% composites at 1000× magnification.

Figure 10 displays the fracture section of the BF/VER composites after the three-point bending test, revealing three failure modes: fiber–matrix interfacial failure, matrix fracture, and fiber fracture. For the control composites, weak interfacial bonding was observed, as evidenced by prominent fiber pull-out and debonding (Figure 10a,b). Additionally, resin-rich regions exhibited smooth fracture surfaces characteristic of brittle failure. In contrast, at a 1.5 wt% KH550-Al_2_O_3_ filler load, the composites showed significantly enhanced fiber–resin bonding. Although localized fiber debonding persisted (Figure 10c,d), the resin adhered tightly to the fractured fibers. Furthermore, the resin-rich regions displayed rougher surfaces, and the matrix demonstrated marked plastic deformation, indicating improved energy absorption during fracture. These observations suggest that the 1.5 wt% KH550-Al_2_O_3_ addition positively influenced both interfacial adhesion and matrix toughness. The experimental results (Section 3.2) confirm that the modified resin matrix exhibits relatively better flexural properties compared to the neat resin. Consequently, the improved flexural properties of the BF/VER composites can be attributed to two synergistic effects: matrix reinforcement through KH550-Al_2_O_3_ modification and strengthened fiber–matrix interfacial bonding.

Specifically, the enhancement of flexural properties in the BF/VER composites by KH550-Al_2_O_3_ primarily stems from synergistic mechanisms, as follows. During the three-point bending tests, the stress distribution within composite specimens exhibits gradient characteristics. When the loading indenter applies downward pressure, maximum compressive and tensile stresses concentrate at the upper and lower surfaces, respectively, while stresses near the neutral axis approach zero. On the tensile-dominated side, KH550-Al_2_O_3_ particles impede microcrack initiation and propagation through a pinning effect within the resin matrix, forcing cracks to circumvent or bifurcate, thereby dissipating energy and dispersing localized stresses. On the compression-dominated side, the high-modulus KH550-Al_2_O_3_ mitigates resin matrix collapse via improved interfacial bonding with the matrix, thus maintaining fiber load-bearing stability. Furthermore, at the fiber–matrix interface, KH550-Al_2_O_3_ facilitates efficient stress transfer to fibers through physical interlocking effects, promoting energy absorption through tending to fiber fracture rather than interfacial debonding. These coordinated mechanisms collectively enhance the flexural properties of the composites.

### 3.4. Dynamic Mechanical Properties of BF/VER Composites

**Figure 11:**
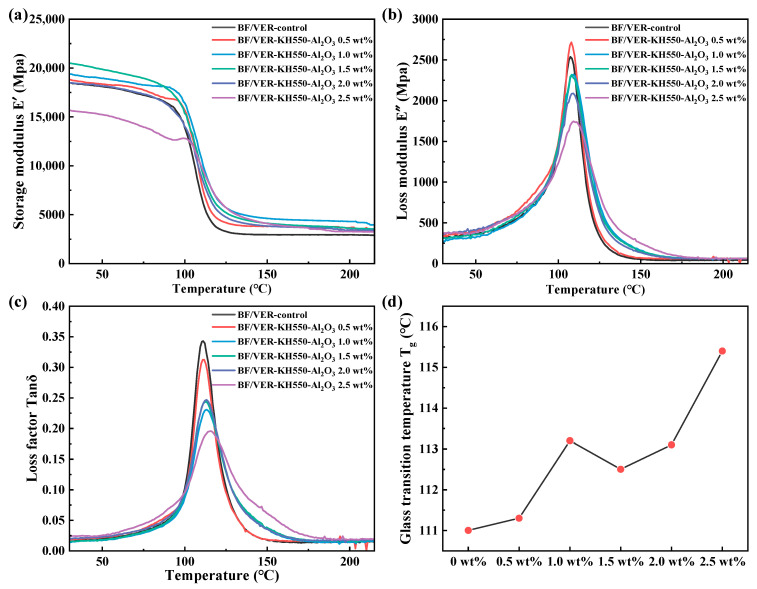
The curves of the (**a**) E′, (**b**) E″, and (**c**) Tanδ as a function of temperature, along with (**d**) T_g_ for the BF/VER composites with different filler loads of KH550-Al_2_O_3_.

Figure 11 presents the temperature-dependent curves of different DMA characteristic parameters for the BF/VER composites with different KH550-Al_2_O_3_ filler loads, measured under the three-point bending mode. The storage modulus (E’) of the BF/VER composites in the glassy region displayed an initial rise followed by a gradual reduction with an increasing KH550-Al_2_O_3_ filler load, as evidenced in Figure 11a. At 1.5 wt% filler load, the storage modulus at 30 °C increased by 11.5% compared with the control composites. Additionally, the modified composites exhibited enhanced storage modulus at elevated temperatures, indicating improved structural stability under thermal loading. This increase in storage modulus demonstrates that KH550-Al_2_O_3_ reinforcement strengthens the load-bearing capacity and stiffness of the composites, effectively reducing deformation under mechanical stress.

The peak loss modulus (E″) of the BF/VER composites exhibited a decline at a KH550-Al_2_O_3_ filler load exceeding 0.5 wt%, as demonstrated in Figure 11b. This reduction in the peak loss modulus indicates that adding KH550-Al_2_O_3_ reduced slip and friction within the BF/VER composites, allowing the fibers and matrix to deform synergistically with improved interfacial bonding. However, at 2.5 wt% KH550-Al_2_O_3_ filler load, the BF/VER composites manifested a marked reduction in the storage modulus in the glassy region, while the peak loss modulus was minimized. This indicates that the increased agglomeration caused by excess fillers led to a simultaneous decrease in the structural rigidity and energy dissipation capacity of the BF/VER composites, which ultimately exhibited poor viscoelastic behavior under dynamic loading.

The loss factor (Tanδ), defined as the dimensionless quotient of loss modulus to storage modulus, is a more reliable parameter for characterizing energy dissipation and storage behavior of material than the loss modulus alone due to its geometry-independent nature [62]. As shown in Figure 11c,d, the peak loss factor of the BF/VER composites with KH550-Al_2_O_3_ addition was reduced and shifted to the right. This decrease in the peak loss factor indicates that the presence of KH550-Al_2_O_3_ made the BF/VER composites more capable of storing externally applied energy, rather than dissipating it as thermal energy. Additionally, since the temperature at which the loss factor reaches its maximum value is commonly employed to identify the T_g_ of the material [63], the rightward shift in the loss factor peak position suggests a rise in the T_g_ of the BF/VER composites. At 1.5 wt% KH550-Al_2_O_3_ filler loading, the T_g_ of the BF/VER composites increased from 111.0 °C to 112.5 °C. This increase stemmed from the presence of KH550-Al_2_O_3_, which constrained the mobility of VER molecular chain segments, reducing the chain migration rate and resulting in a higher T_g_ for the composites.

While seemingly modest, the observed increase in the T_g_ of the composites holds significant practical implications for industrial and engineering applications. Laminated fiber-reinforced resin matrix composites exhibit distinct failure modes at different temperatures. When the temperature is significantly below their T_g_, the composites demonstrate resin matrix-dominated brittle fracture behavior accompanied by fiber fracture [64]. As the temperature approaches or exceeds T_g_, the resin matrix transitions between glassy and rubbery states, resulting in material softening. This leads to extensive fiber–resin interfacial debonding and a significant decline in the mechanical properties of the composites [65]. In high-performance fiber-reinforced polymer (FRP) composites, even a slight improvement in T_g_ is directly related to the enhancement of thermal stability and load-bearing capacity at elevated temperatures, as the heat resistance of the resin matrix directly affects the heat resistance of the composites [66]. For FRP-strengthened reinforced concrete members with good structure, the T_g_ is the threshold for the occurrence of fire in the composites [67]. For conventional FRP composites, the T_g_ directly determines their actual operating temperature, especially for aerospace applications [68]. The higher T_g_ ensures that the material retains its structural integrity and stiffness closer to its operational temperature limits, which minimizes the risk of premature failure under sustained loads, thereby extending the service life of critical parts.

### 3.5. Flexural Properties of BF/VER Composites After Long-Term Elevated Temperature Aging

**Figure 12:**
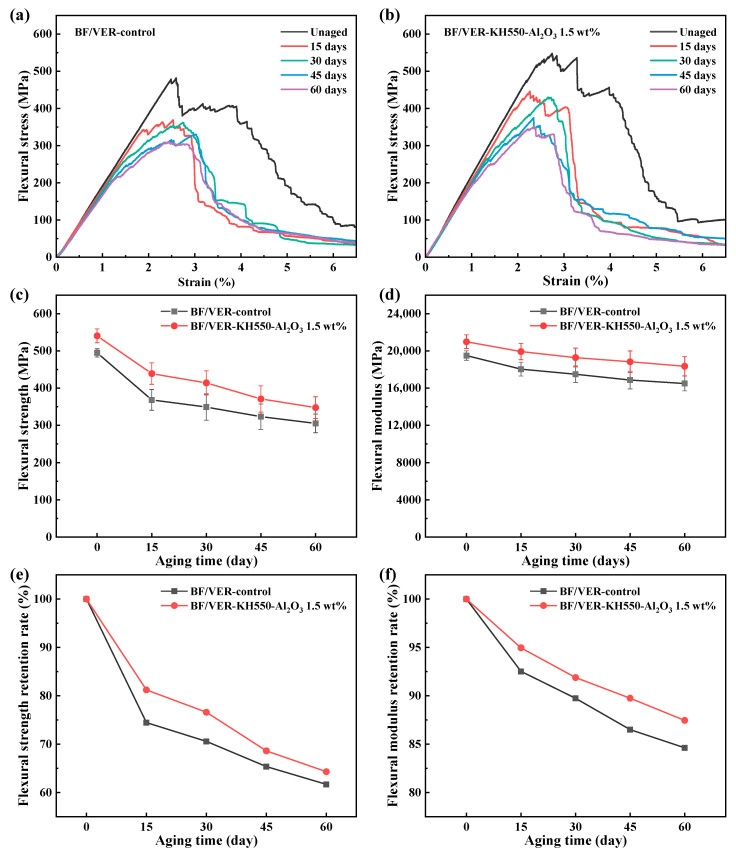
Flexural properties of the BF/VER composites following thermal aging at 120 °C for varying durations: (**a**) the control BF/VER composites’ flexural stress–strain curves, (**b**) the BF/VER-KH550-Al_2_O_3_ 1.5 wt% composites’ addition flexural stress–strain curves, (**c**) flexural strength, (**d**) flexural modulus, (**e**) flexural strength retention rate, and (**f**) flexural modulus retention rate.

Figure 12 presents the flexural properties of the BF/VER composites following long-term thermal aging at 120 °C for varying durations. As shown in Figure 12a,b, regardless of whether fillers were added, the area beneath the flexural stress–strain curves of all the composites after aging decreased significantly and the peak stress was reduced, indicating that the composites, after elevated temperature aging, were less able to absorb energy under bending load and became more brittle, leading to a marked reduction in load-bearing capacity. By 30 days, the slope within the linear elastic region of the stress curve significantly decreased, while the stress exhibited a steep upward slope (with a pronounced gradient) beyond the linear elastic region. This indicates that the resin matrix subjected to prolonged thermal aging can no longer effectively transfer loads to the fibers, leading to a significant degradation in the flexural properties of the composite materials. As shown in Figure 12c–f, the flexural properties of both the composites demonstrated progressive deterioration with prolonged aging duration, manifested as a significant decrease in flexural properties after 15 days of aging time, and the decrease slowed down slightly after this aging stage. At 1.5 wt% KH550-Al_2_O_3_ filler loading, the BF/VER composites exhibited better retention of flexural properties after long-term elevated temperature aging compared with the control BF/VER composites, with the flexural strength and flexural modulus retention rates after 60 days of aging at 64.3% and 87.4%, respectively.

During the initial aging phase (less than 15 days), the resin matrix undergoes progressive post-curing, accompanied by an elevation in cross-linking density. However, excessive cross-linking induces embrittlement of the matrix due to restricted segmental motion [18]. With prolonged aging, thermo-oxidative degradation dominates, disrupting the native molecular architecture of the resin. Concurrently, volatiles released from resin decomposition contribute to increased porosity and defects within the material. The growing disparity in mechanical properties between the resin matrix and reinforcing fibers amplifies thermally induced stress concentrations. This culminates in progressive deterioration of the fiber–matrix interfacial integrity, manifesting as a marked decline in the flexural properties of the composites. As aging continues (more than 15 days), a considerable portion of the residual stress inside the composite material is released, and the degradation of fiber properties usually gradually becomes apparent after a longer period. At this stage, the degradation of the composite properties is primarily caused by slow oxidation reactions, leading to a gradual and gradual decline in the subsequent properties.

The BF/VER-KH550-Al_2_O_3_ 1.5 wt% composites exhibit better flexural properties retention during long-term elevated temperature aging, which may be attributed to two factors. On the one hand, as discussed in previous sections, KH550-Al_2_O_3_ exhibits reinforcing effects on both the resin matrix and the composites. The chain movement of resin under elevated temperatures is effectively constrained by KH550-Al_2_O_3_, thereby maintaining stress transfer efficiency and mitigating interfacial debonding in the composites. On the other hand, the high thermal conductivity of KH550-Al_2_O_3_ facilitates heat dissipation while simultaneously acting as a barrier against oxygen permeation. These combined effects contribute to enhanced stability of the composites at elevated temperatures.

**Figure 13:**
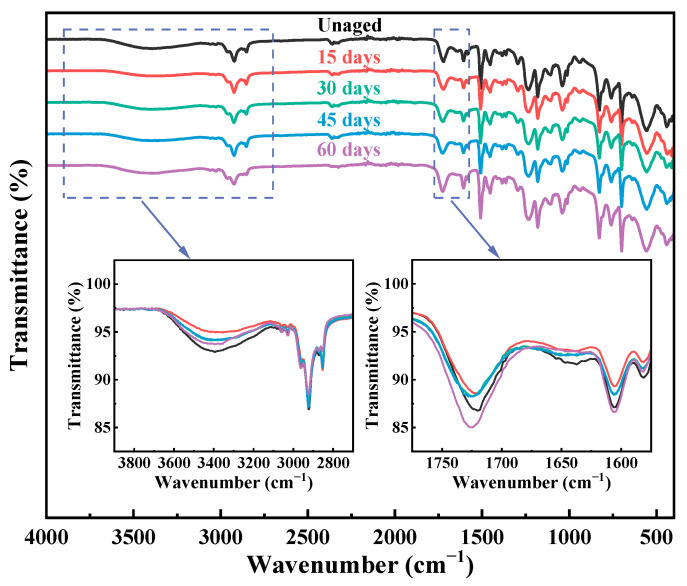
The infrared spectra of the BF/VER-KH550-Al_2_O_3_ 1.5 wt% composites after aging.

Figure 13 displays the infrared spectra of the BF/VER-KH550-Al_2_O_3_ 1.5 wt% composites after aging at 120 °C for varying durations. The composites exhibit complex spectral evolution behavior after elevated temperature aging. The reduction in the stretching vibration absorption peak of C=C bonds at 1640 cm^−1^ demonstrates the continuation of cross-linking reactions in the VER under elevated temperatures, leading to resin embrittlement. Concurrently, the significant decrease in the O-H stretching vibration absorption peak at 3400 cm^−1^ after 15 days of aging corroborates the consumption of free hydroxyl groups due to further cross-linking. As aging progresses, the O-H peak intensity partially recovers but remains lower than that of the unaged sample, indicating that the resin does not undergo complete degradation in later aging stages. Instead, molecular chain scission induced by oxidation reactions and hydrolysis gradually dominates. The overall attenuation of C-H bond stretching vibration absorption peaks between 2970 and 2840 cm^−1^ suggests the VER degradation. The weakening of the C=O stretching vibration absorption peak at 1722 cm^−1^ after 15 days of aging is attributed to ester group hydrolysis. However, the C=O peak intensity gradually increases with prolonged aging time. Notably, after 60 days of aging, its intensity surpasses that of the unaged sample. Since VER curing primarily relies on radical copolymerization between C=C bonds in its molecular chains and styrene monomers, no direct evidence supports the formation of carbonyl-containing compounds from styrene via thermal oxidation [69]. Therefore, the enhanced C=O peak implies the generation of new carboxylic acids or ester compounds through the thermal oxidation of the VER itself. The changes in the characteristic absorption peaks mentioned in the appeal collectively confirmed undergoing degradation due to thermal oxidation of the VER matrix in the composites.

**Figure 14:**
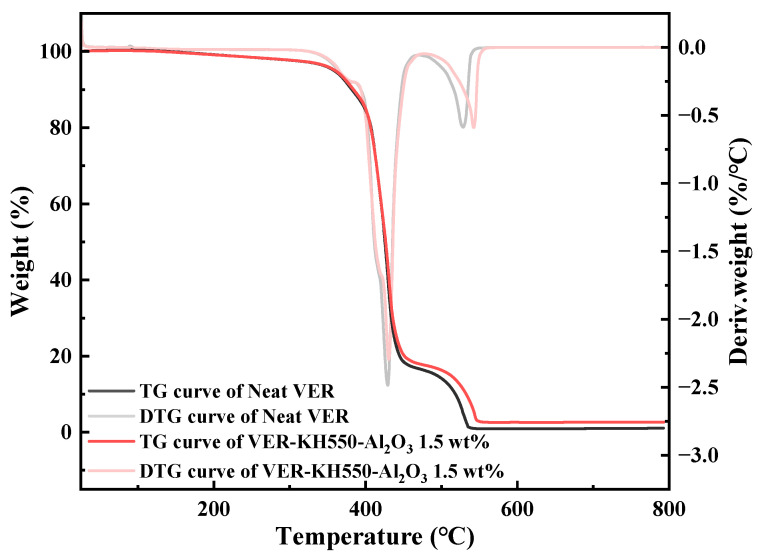
TGA curves of the neat VER and the VER-KH550-Al_2_O_3_ 1.5 wt% resin in air.

Additionally, as shown in Figure 14, the TG and DTG curves of the neat VER and the VER-KH550-Al_2_O_3_ 1.5 wt% resin in the air are nearly identical, confirming that the presence of KH550-Al_2_O_3_ maintained the thermal stability of the VER. Meanwhile, BF does not decompose significantly below 200 °C [70]. Consequently, the reduced flexural properties of the BF/VER composites after long-term elevated temperature aging were attributed to both matrix degradation caused by thermal oxidation and the physical aging phenomenon.

**Figure 15:**
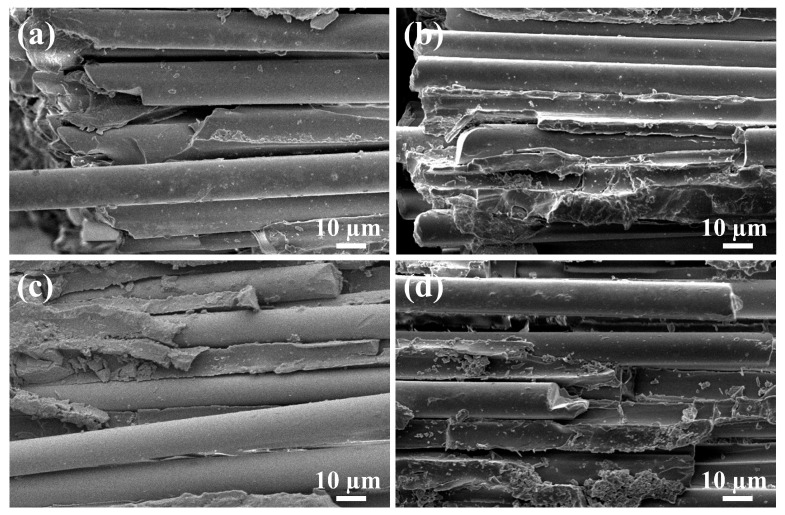
SEM images of the fracture section of BF/VER composites after elevated-temperature aging: (**a**) the control BF/VER composites aged for 15 days, (**b**) the BF/VER-KH550-Al_2_O_3_ 1.5 wt% composites aged for 15 days, (**c**) the control BF/VER composites aged for 60 days, and (**d**) the BF/VER-KH550-Al_2_O_3_ 1.5 wt% composites aged for 60 days.

Figure 15 compares the fracture surfaces of the control BF/VER composites and the BF/VER-KH550-Al_2_O_3_ 1.5 wt% composites after aging at 120 °C for 15 and 60 days. After 15 days, both groups exhibited exacerbated interfacial debonding and elongated fiber pull-out lengths (Figure 15a,b). In contrast, the modified composites retained marginally superior fiber–resin bonding. By day 60, the interfacial debonding had worsened further, accompanied by severe delamination in both groups of composites (Figure 15c,d). These observations suggest that while KH550-Al_2_O_3_ enhances interfacial adhesion during early-stage aging, prolonged exposure inevitably degrades interfacial properties due to physical aging and thermal oxidation-induced matrix degradation.

## 4. Conclusions

This study utilized surface-modified nano-Al_2_O_3_ treated with the silane coupling agent KH550 to modify VER and prepare the BF/VER composites. The effect of KH550-Al_2_O_3_ on the properties of the BF/VER composites was investigated, including flexural properties, viscoelastic behavior, and flexural properties after long-term elevated temperature aging. The following conclusions were drawn:

- Silane coupling agent KH550 surface modification can effectively reduce the agglomeration of nano-Al_2_O_3_ in the VER, but the formation of excessive polysiloxane weakens the positive effect of surface modification. The main effects of whether or not nano-Al_2_O_3_ was modified and the effects of its loading on the flexural properties of VER were statistically significant. Still, the interaction between the two factors did not reach the level of significance for the flexural strength or flexural modulus. Unmodified nano-Al_2_O_3_ and KH550-Al_2_O_3_ enhanced the flexural properties of the VER, primarily through crack deflection and crack pinning mechanisms. At 1.5 wt% KH550-Al_2_O_3_ filler loading, the VER attained peak flexural strength and modulus values, showing 18.1% and 22.7% increases, respectively.
- The flexural properties of BF/VER composites were enhanced by both matrix reinforcement and improved fiber–resin interfacial bonding. At 1.5 wt% KH550-Al_2_O_3_ filler loading, the BF/VER composites attained peak flexural strength and modulus values, showing 9.3% and 7.6% increases, respectively.
- Adding a certain amount of KH550-Al_2_O_3_ increased the storage modulus and T_g_ of the BF/VER composites but reduced the peak loss modulus. At 1.5 wt% KH550-Al_2_O_3_ filler loading, the storage modulus at 30 °C improved by 11.5%, while T_g_ increased from 111.0 °C to 112.5 °C.
- The addition of 1.5 wt% KH550-Al_2_O_3_ enabled the BF/VER composites to maintain higher flexural properties during long-term elevated temperature aging at 120 °C. The thermal stability of the VER remained unaffected, with the property degradation of the BF/VER composites attributed to both matrix degradation caused by thermal oxidation and the physical aging phenomenon. After 60 days of aging, the flexural strength and modulus retention rates of the composites were 64.3% and 87.4% of those before aging, respectively.

## Figures and Tables

**Table 1:** Two-way ANOVA test results for the flexural strength of the modified resin.

Source	Type III Sum of Squares	df	Mean Square	F	Sig.
Corrected Model	1234.752	9	137.195	6.289	0.000
Intercept	683,949.332	1	683,949.332	31,354.057	0.000
Modified or unmodified	292.626	1	292.626	13.415	0.001
Filler loading	860.335	4	215.084	9.860	0.000
Modified or unmodified × Filler loading	81.790	4	20.448	0.937	0.452
Error	872.550	40	21.814		
Total	686,056.633	50			
Corrected Total	2107.302	49			

**Table 2:** Two-way ANOVA test results for the flexural modulus of the modified resin.

Source	Type III Sum of Squares	df	Mean Square	F	Sig.
Corrected Model	862,628.222	9	95,847.580	4.862	0.000
Intercept	532,072,910.5	1	532,072,910.5	26,991.195	0.000
Modified or unmodified	425,230.109	1	425,230.109	21.571	0.000
Filler loading	259,792.559	4	64,948.140	3.295	0.020
Modified or unmodified × Filler loading	177,605.554	4	44,401.389	2.252	0.080
Error	788,513.302	40	19,712.833		
Total	533,724,052.1	50			
Corrected Total	1,651,141.524	49			

## Data Availability

The original contributions presented in this study are included in the article. Further inquiries can be directed to the corresponding author.
